# Supplementary material for: Are carbon emissions trading and green financial instruments synergistic? -Comprehensive quantitative research based on content analysis
Source: PLoS One. 2024 Mar 7;19(3):e0298601. doi: 10.1371/journal.pone.0298601 (PMC10919601; doi:10.1371/journal.pone.0298601)
Supplement: S1 Data — (ZIP) [file pone.0298601.s001.zip › Data/地级市Z_F工作报告.zip!/║┌┴·╜¡/╝a╬≈╩╨/└┤╫╘╣1⁄2╓┌║┼í╛┬φ┐╦╩2╛▌═°í┐_2014.docx]

各位代表：

现在，我代表市政府，向大会作工作报告，请予审议。

一、2013年工作回顾

2013年，是极不平凡的一年。面对经济下行的严峻考验，在市委的坚强领导下，在市人大、市政协的监督和支持下，市政府与全市人民知难而上，共克时艰，经济社会保持了持续发展的势头。全市地区生产总值实现571亿元，增长0.9%；规模以上工业增加值100亿元，下降6.4% ；公共财政预算收入33.4亿元，下降22.7%；固定资产投资299.8亿元，增长8%；社会消费品零售总额184.4亿元，增长13.7%；外贸进出口总额12亿美元，增长11.3%；城镇居民人均可支配收入17697元，增长4.2%；农民人均纯收入11985元，增长15%。

——项目建设进展顺利。全年共实施投资千万元以上产业项目、民生及基础设施项目291个，完成投资175亿元。鸡西义乌国际商贸城投入运营，珍宝岛药业中成药、万达鸡西商贸中心等一批项目开工建设，为鸡西长远发展奠定了基础。对上争取成效显著，我市申报全国老工业基地调整改造试点城市等项目取得突破性进展，兴凯湖参加国家良好湖泊保护项目竞争立项成功，全年共争取到位资金62亿元，是鸡西历史上争取项目最多、得到支持最大的一年。园区建设提档升级，市工业示范基地成功晋升省级经济开发区。

——三次产业协调发展。一、二、三产比重为29.2：38.5：32.3。“三农”工作稳步推进。粮食生产再攀新高，总产量达69.1亿斤，增长6.3%，增幅居全省第2位。全市绿色食品种植基地监测面积达467万亩，新增市级以上龙头企业6户。工业经济逆势前行。举办了地方煤矿和煤机企业供销对接会，签订合作协议资金1.1亿元。召开了银企对接会，为企业贷款66.4亿元。出台了《鸡西市工业裂变升级实施方案》，全力推动企业转型升级、扩张发展。第三产业繁荣活跃。投资4.6亿元实施了兴凯湖湿地公园等20个旅游项目，旅游业总收入31.6亿元，增长12.4%。积极创新融资模式，多方筹集资金28.9亿元，增长45%。金融机构累计投放贷款356.7亿元，增长15.2%。

——改革开放日益深化。全市行政审批事项由579项精简到409项；实施了城建项目并联审批和公交改革；密山、虎林被列为全省“两大平原”现代农业综合配套改革重点县(市)。密山、虎林口岸开展了异地办证业务，新开通了鸡西—伊曼国际货运线路、鸡西—沈阳—三亚航线，对外联系更加顺畅。全年引进国内到位资金229.6亿元，增长30.9%；实际利用外资1.2亿美元，增长20%。

——城市功能不断完善。全市人民翘首企盼的城市供水工程获省政府批准，困扰城市多年的燃气工程开工建设，市民反映强烈的城市供热问题得到较好解决，满意度不断提升。改扩建红旗路等17条道路，开工建设东山桥公园，全市新增绿地面积145公顷。鸡冠新区建设步伐加快，4条主要街路实现全线通车，体育会展中心投入使用。

——民生建设成果丰硕。全年财政共投入民生资金46.4亿元，增长29.2%，占财政总支出的34.4%，向人民承诺的30件民生实事件件落实。保障性安居工程超额完成目标任务，新开工6391套，竣工14333套，发放廉租住房补贴6000余万元。

——社会事业全面进步。新建续建校舍7万平方米，鸡西大学顺利升本，市医院新址开工建设，全市65家博物馆、图书馆、文化馆(站)免费开放，鸡西石墨产业技术创新战略联盟晋升为国家级试点联盟。成功举办了国奥男篮四国挑战赛，扩大了鸡西的对外影响力。强化人才柔性引进机制，“兴凯湖智力行”活动成效显著。社会更加安全稳定，全市安全事故减少48.9%，重特大刑事案件发案下降36.2%。

提前介入，超常规运作，积极稳妥处理北钢问题，切实维护了广大职工合法权益。

各位代表，回顾一年来的艰辛历程，我们深感成绩来之不易，这是省委、省政府和市委正确领导的结果，是市人大、市政协和社会各界大力支持的结果，是全市人民和衷共济、拼搏进取的结果。在此，我代表市政府向全市广大干部群众，向所有关心和支持鸡西发展的同志们、朋友们，表示衷心的感谢，致以崇高的敬意!

我们也清醒地认识到，我市经济社会发展中还存在不少困难和问题。受宏观经济下行、煤炭市场持续低迷的影响，煤炭产业效益大幅下滑，导致部分经济指标没有完成预期；体制性和结构性矛盾长期存在，产业结构单一、产品附加值低、企业竞争力不强的问题仍然突出；立市、立县(市)区的大项目、好项目少，部分在建项目进展迟缓；财政收入增速放缓，刚性支出压力大，公共服务、民生改善与广大群众的期盼还有一定差距；安全生产和煤矿整合的压力还很大；机关工作作风还需大力改进，发展环境还需进一步优化，等等。对以上困难和问题，我们绝不回避，勇于面对，并将采取有力措施，切实加以解决。

二、2014年工作安排

2014年，政府工作的总体要求是：深入贯彻落实党的十八届三中全会精神，按照省委十一届四次全会、市委十二届四次全会部署，紧紧抓住省实施“五大规划”的战略机遇，以改革创新为统领，以科学发展、绿色发展为主题，以稳中求进、进中求好为主基调，坚持上项目增实力，抓发展惠民生，保稳定促和谐，力求在调整结构、转变方式上有新进展，在提高质量、增强效益上有新突破，在备足后劲、打牢基础上有新成效，开创鸡西又好又快发展新局面，努力让人民群众生活得更加美好。

经济社会发展的主要预期目标是：地区生产总值增长8%左右，规模以上工业增加值增长7.5%以上，固定资产投资增长25%左右，公共财政预算收入增长8%左右，社会消费品零售总额增长13.5%左右，外贸进出口总额增长8%，城镇居民人均可支配收入和农民人均纯收入均增长10%以上。节能减排指标达到省核定标准。

为实现上述目标，重点抓好七个方面工作。

(一)以项目建设为牵动，打造经济发展新引擎

项目建设是立市之本、经济之根、民生之源、稳定之基，必须举全市之力、动员域内一切力量，切实抓紧抓好、抓出成效。

强力推进项目建设。重点实施投资5000万元以上产业项目141项，总投资415.6亿元，当年完成投资171.7亿元，增长30%以上。开工建设投资10亿元的招华物流配送、6亿元的青岛啤酒异地扩建等112个新项目；加快推进投资14亿元的虎头旅游名镇、13.5亿元的山东泰丰煤矿改造等29个续建项目。完成投资6亿元的福娃食品产业园、2亿元的贝特瑞石墨扩建等68个项目建设任务。

做大做强产业园区。坚持规划引领，推进政策优先向园区倾斜、土地优先向园区集中、项目优先向园区摆放。重点建设规划面积40平方公里的市煤化工产业园区，先期启动3平方公里核心区，完成“七通一平”，重点引进建设煤制油、煤制烯烃等大型煤化工项目。鸡西(鸡冠)产业园区，完成土地收储133公顷，大上食品医药、机械电子、汽车组装等产业项目，着力打造综合工业园区。恒山和麻山石墨产业园区，加快引进建设一批石墨精深加工及高附加值配套项目，着力打造石墨新材料产业基地。梨树工业谷，加大基础设施建设力度，围绕煤炭综合利用、镁合金精深加工等方向，谋划建设一批重点项目，着力打造资源综合利用示范区。创新园区管理体制，优化职能配置，形成推进合力。活化“飞地经济”模式，科学配置资源，促进产业集聚。

全力以赴招商引资。建立“谋划、招商、建设”链条式项目推进机制，紧紧围绕政策导向、资源优势、重点产业，与大专院校、科研院所深度合作，加大投入，谋划储备一批立市、立县(市)区的大项目、好项目。充分发挥政府引导和企业主体的作用，整合力量，紧盯世界500强、央企国企、大型民企，做好赴“长三角”等地的定向招商、定点招商和专题招商活动，积极参加省组织的港粤经贸交流和中国俄罗斯博览会等活动，大力引进战略投资者、行业领军企业和高新技术企业。全年引进国内到位资金增长20%，实际利用外资增长10%以上。

千方百计对上争取。密切关注国家和省政策导向、资金扶持领域，做实基础，主动出击，集中获取政策红利。力争将滴道大同沟独立工矿区纳入国家老工业区搬迁改造范围，《兴凯湖流域保护综合规划》获得国家批复；全力抓好国有工矿棚户区、城市供热管网改造等项目资金的争取工作。

(二)以转型升级为导向，构筑工业经济新优势

工业的“脊梁”不硬，财政的“身板”就挺不起来。大力实施“双轮驱动”战略，引进增量与做大存量并举，扩张总量与提升质量并重，全面提高工业经济综合实力。

全力实施“两黑一绿”产业集群战略。充分发挥市场配置资源的决定性作用，强化要素保障，全力构建立市支柱产业群。现代煤化工产业群，以推进煤炭深加工为方向，着力发展延伸煤制甲醇、煤制芳烃等产业链，重点建设宝泰隆45万吨煤矿联产20万吨芳烃、龙昌硅藻土开发等20个煤化工项目。石墨精深加工产业群，围绕构建密封材料、超硬材料、传导材料等七大产业链，重点推进普莱德锂电池负极材料、贝特瑞新型球形石墨、瑞诺恒金刚石二期等26个深加工项目。整合石墨资源，增强调控权、配置权。绿色食品及生物医药加工产业群，瞄准功能食品、休闲食品、保健食品等市场需求，重点推进汇源集团浆果深加工、益海嘉里水稻综合加工、珍宝岛药业穿心莲碱D1等23个项目。

大力实施企业裂变升级战略。以打造“百亿航母、十亿板块、亿元群体”为目标，鼓励支持域内企业引入发展要素、优化资源配置，推动企业延伸发展、扩张发展、规模发展。围绕升级式裂变，重点推进东北王酒业20万吨饮用水、赛龙水泥日产2500吨熟料等22个技改项目。围绕扩张式裂变，重点推进天盛石墨深加工、山东泰丰120万吨洗煤等28个新项目。围绕合作式裂变，重点推进兴凯湖集团大豆植物蛋白乳加工、完达山酒业万吨白酒等15个合作项目。制定出台支持企业“裂变升级”的具体措施，支持具备条件的小微企业在创业板上市融资。

(三)以富民增收为主线，开创“三农”工作新局面

紧紧抓住“两大平原”现代农业综合配套改革试验的战略机遇，坚持用工业化理念和市场化思维发展现代农业，着力推动种植业向畜牧业战略转变，传统农业向绿色品牌农业战略转变。

大力发展畜牧业。围绕发展“两牛一猪”重点产业，大力引进先进技术、优良品种和管理经验，培育养殖大户，实现畜牧业养殖规模化、标准化。坚持“四统一”(统一规划、统一饲养、统一防疫、统一管理)，积极推进畜禽养殖“三退三进”(退出散养、进入大户，退出庭院、进入牧场，退出村屯、进入小区)，新建万头牛场1处、千头牛场5处、万头猪场7处，创建市级标准化养殖场20处、畜牧小区10处。坚持龙头带基地，基地联农户，扶持完达山乳业、娃哈哈集团等龙头企业发展壮大，加快建设天翔牧业肉牛屠宰等一批肉类加工项目。全年畜牧业产值占农业总产值25%以上。

大力发展绿色品牌农业。进一步扩大绿色食品种植基地规模，监测面积达495万亩，绿色、有机、无公害农产品标识达146个。突出龙头带动作用，做大做强绿都、中粮、东粮、雨润等龙头企业，积极引进大型食品深加工企业，市级以上农业产业化龙头企业发展到85户。整合域内品牌资源，集中叫响大米、乳品、肉类、蜂蜜、湖鱼等绿色农产品、优势畜产品、特色山产品、名优水产品品牌，争创一批驰名、著名商标和地理标志商标，以品牌促整合、以品牌闯市场、以品牌带增收。围绕绿色食品生产，进一步加强水利设施建设，加快先进农机具普及应用、先进农业技术推广。推进农业经营方式创新，依法依规进行土地流转。

(四)以旅游贸易为重点，强化第三产业新支撑

着眼域内域外两大市场，面向生产生活两大需求，着力提升第三产业发展规模和水平，增强对经济发展的拉动能力。

做大旅游业。编制完成《鸡西市旅游发展总体规划》，全力做好旅游“延点、延线、延时”三篇文章。加强景区景点建设，积极推进兴凯湖旅游带综合开发、虎头旅游名镇、鸡东凤凰山生态旅游等重点项目建设，开工建设密兴高速公路，启动实施虎头国家5A级景区创建工作。延伸旅游线路，以兴凯湖、虎头景区为核心，整合域内资源，做优生态休闲游、观光避暑游、养生度假游、历史文化游等旅游线路；利用异地办证政策优势，大力发展对俄跨国游；加强与域外旅游名城、鸡西友好城市、国内知名旅行社的合作，推进线路对接。深度开发“春观花鸟、夏游江湖、秋看山水、冬赏冰雪”四季旅游精品，推动旅游业由一季繁荣向四季兴旺转变。充分利用各类媒体，加大宣传力度，让更多外地游客了解鸡西、走进鸡西。全年旅游业总收入增长10%以上。

做活外贸产业。依托密山、虎林两个口岸，借助国内国外两个市场，内建工厂、外建基地，内引外联，发展口岸经济。加快推进密山口岸、鸡西海关监管仓库建设，完善口岸功能。发挥鸡西至俄罗斯客货运输通道作用，吸引人流、物流、资金流向口岸聚集流动。坚持“出口抓加工、进口抓落地”，进一步加强密山、虎林进出口加工园区建设，引进上海安信地板等企业入驻，支持日信食品、家利利等企业扩张发展，提高远华、茂盛公司就地加工比例，新增加工型外贸企业5户；扶持在俄企业建设生产加工原料基地，开展粮食回运和木材加工业务。全年地工产品出口增长20%以上，进口资源落地加工增长30%以上。

做强商贸物流业。加快万达鸡西商贸中心等项目建设，支持大商新玛特等大中型商贸企业做大做强。发挥义乌国际商贸城的品牌效应，建设黑龙江东部及远东地区小商品集散中心。巩固提升煤炭、粮食等主体物流，积极培育速递配送、冷链储运和第三方物流业态。加快推进义乌仓储配送中心、招华物流配送中心等现代物流项目建设，加强农产品集散、生产资料交易、汽车综合服务等市场建设，构建现代物流体系。

大力发展金融、会展等现代服务业。加大域外金融机构引进力度，支持民间资本进入小额贷款和融资担保行业。做好企业债券发行，推进城市供水、保障房建设等项目融资。完成鸡西市区农村信用社改制工作。加强金融监管，优化金融生态环境。大力发展会展经济，谋划办好黑龙江东部地区绿色食品交易会、石墨新材料产品展示会、中俄义乌小商品交易会等展会，打造鸡西展会品牌。积极发展文化创意、时尚消费、电子商务、居家养老、休闲健身等新兴服务业。

(五)以宜居宜业为目标，推动城乡面貌新改观

围绕打造以鸡冠区为中心，鸡东、密山、虎林为副中心的“一核三点”城市圈，坚持“布局合理、功能配套、特色鲜明”的原则，强力推进“四区一县城”同城化步伐，着力提升城市综合服务功能。

坚持规划引领。启动第四轮城市总体规划修编，编制“四区一县城”、滨水城市建设等概念性规划，同步编制棚户区改造、供热、供气、供水等专项规划。结合土地中期评估，完成300公顷控制性详细规划和100公顷修建性详细规划。

大力改造老城区。坚持“增空间、增绿地，减高层、减容积率”，实施五大工程，提升老城区功能。路桥建设工程。开工建设穆棱河大桥等4座桥梁，拓宽鸡兴西路，改扩建富强路中段等21条主要街路。管网改造工程。推进城市供水配套工程建设；新建、改造供热管网57公里，建设换热站10座，新增供热能力220万平方米；开工建设城市西部排水干管和培新路排水工程。绿化亮化工程。开工建设城市森林公园，实施南星街绿化景观带建设，完成康乐街等4条街路绿化；推进背街巷道和居民小区亮化，完成既有建筑节能改造10万平方米。配套设施建设工程。新建红军路、东胜街等10处停车场；加快推进万达广场中心大街地下人防等续建项目。滨水城市建设工程。结合穆棱河综合治理，加快土地收储，完善基础设施，开发穆棱河北岸，打造“一河居中、两岸同兴”发展新格局。

加快建设新城区。完成康新路、长征街等8条街路绿化和长胜路亮化；推进九粮路等主干街路与老城区贯通。加快商业综合体、旅游地产、会展等项目建设，带动商贸、物流、房地产等产业发展。

打造特色小城镇。制定《城镇化发展规划》，科学布局城镇空间、产业、人口、公共服务，重点推进连珠山镇、向阳镇、虎头镇等一批工业重镇、商贸集镇、旅游名镇建设。发挥农垦、森工带动作用，合作共建裴德镇、东方红镇等特色小城镇。大力推进美丽乡村建设，实施村屯亮化绿化美化，加强环境综合整治。

强化城市管理。实施“大城管”机制，加大公安、城管、环保、环卫等部门联合执法力度。投入2660万元，更新清雪等环卫设备，提高机械化作业水平。启动数字城市建设系统，加强市政设施的精准监控和日常维护。开展环境卫生、低空噪音、私搭乱建、占道经营、牌匾广告、乱贴乱画等专项整治行动，对违规违法行为严管严罚。建立健全市民参与城市管理的体制机制，营造“人民城市人民建、人民城市人民管”的良好氛围。

(六)以改善民生为根本，推进社会事业新进步

坚持新增财力向民生投入、工作力量向民生倾斜，重点办好群众最关心的11件实事。(1)城市供水问题已困扰我市多年。我们将全力推进投资15亿元的城市供水工程建设，今年3月份开工建设，两年时间建成，2016年3月份让我们的市民喝上清冽甘甜优质的放心水、健康水。(2)燃气工程一头连着政府的心，一头连着百姓的厨房。我们与中国燃气公司合作，投资4500万元，建设临时燃气厂站1座、永久性燃气厂站2座，并且免费改造居民炉具，让广大市民用上优质、高效、安全的清洁能源。(3)为了让城市交通大动脉更加畅通，让群众出行更加快捷，今年我们将开工建设穆棱河大桥、团结立交桥、左家立交桥、滴道公铁立交桥四座大桥。(4)解决安居问题，既是群众所盼，也是政府面临的一项长期的、艰巨的、紧迫的任务，不管多难也要办好。我们将加快保障性安居工程建设，开工4673套、竣工5050套，让人民群众住得好、住得暖、住得舒心。(5)把鸡西建设成为美好家园，是我们共同的追求和梦想。今年将建成西山城市森林公园、东山桥公园，让我们守望相助的家园更宜居、更美丽。(6)众多的背街巷道一直是城市里被遗忘的角落。我们将集中改造背街巷道100条，让广大群众彻底告别泥泞路、搓板路、黑暗路。(7)物业管理是近年来群众反映最强烈的问题之一。今年，将投入5000万元，改造中心区弃管楼50栋，全部达到三级物业服务标准。开展“星级”物业企业评比，重点解决物业管理水平低、小区环境脏乱差的问题，力争80%的小区达到三级以上物业服务标准，让市民享受到等值、超值、高水平、高质量的物业服务。(8)孩子们是家庭的希望，祖国的未来。我们将进一步加大教育投入，新建续建校舍2.3万平方米；完善助学体系，全年资助家庭困难学生3.5万人，让每一个孩子都能上得起学、上得好学。(9)健康是百姓最大的幸福。为了让群众享受更好的医疗服务，我们将着力解决异地就医和医保关系转移接续问题，加强与北京、上海、天津等大城市的名院、名医合作，让群众看病更有保障、更加便捷、更有质量。(10)社区是服务群众的第一窗口。我们将全力推进标准化社区建设，创建12个标准化城乡社区，努力为群众提供最贴心、最周到的就业社保、居家养老、文体娱乐等公共服务，让社区成为群众安居乐业的和谐家园、幸福家园。(11)文化是群众的精神食粮。我们将加快推进文化惠民工程，开工建设市文化艺术中心，推进鸡冠区、恒山区、鸡东县文化馆建设，让广大群众的精神文化生活更丰富、更多彩。

(七)以责任落实为保障，促进安全稳定新提升

安全关乎经济社会发展，关乎社会和谐稳定，关乎人民群众切身利益，必须持之以恒、长抓不懈。

突出抓好煤矿安全。继续推进煤矿整治整合，加快组建煤炭集团公司。加强煤矿监管，严格落实政府属地管理责任、部门监管责任、企业主体责任和职工岗位责任。继续实施“四个专项推进”和“两个专项整治”，突出治理以瓦斯和水患为重点的各类隐患。加大“打非治违”力度，确保非法井始终保持“零状态”。

切实加强地面安全。突出抓好非煤矿山、危险化学品、烟花爆竹等行业安全监管，全力抓好道路交通、消防森防、食品药品、建筑施工等重点领域和商场、学校等人员密集场所的安全生产工作。

努力维护社会稳定。加强和创新社会治理，健全重大决策社会稳定风险评估机制。畅通和规范群众诉求表达渠道，认真解决好人民群众的信访问题。以“转树练”活动为载体，提高公安队伍执法规范化水平，加快推进公安科技信息化建设，创新立体化社会治安防控体系，依法严密防范和惩治各类违法犯罪，让群众生活的更有安全感。切实提升应急救援能　　三、切实加强改革和政府自身建设

面对发展的新机遇、改革的新要求、人民群众的新期盼，政府使命在肩、责任重大。我们将以改革创新统领政府各项工作，不断提高推动科学发展的能力和水平。

大力推进改革。按照国家和省部署，正确、准确、有序、协调推进各项改革，让改革充分释放红利。深化行政审批制度改革，继续取消和下放行政审批事项，坚决纠正和取消没有上位法许可和变相保留的审批项目。建立健全项目建设“并联审批”、“秋冬审批”工作机制；推行网上审批，完成网上政务服务中心建设。实施财税体制改革，加快推进部门预算改革，编制政府性基金预算和社保基金预算，强化预算约束。完善国库集中收付制度，实现各类财政性资金支付过程信息化、动态化管理。加快公务卡改革扩面。分步推进预决算公开。稳妥推进机构改革，做好工商、质监等省垂直管理部门职责划转地方承接工作，推进人口计生与卫生部门的机构改革合并。加快推进事业单位分类改革，按照省统一部署，完成事业单位分类，适时开展事业单位“十定”工作。继续推进公交线路改革。

依法依规办事。坚持科学民主决策，进一步完善公众参与、专家论证和集体讨论相结合的决策制度，强化事前控制、事中监督、事后评估，不断提高政府决策的规范性和科学性。坚持依法行政，严格按照法定权限和法定程序行使权力，自觉接受人大法律监督、政协民主监督，认真办理人大代表议案、建议和政协委员提案，虚心听取民主党派、工商联、无党派人士和各人民团体的意见。更加重视社会监督和新闻媒体监督，继续完善政务公开、新闻发布、社会公示和听证制度，增强政府工作透明度。采取积极有效措施，切实改善发展环境，着力打造投资“洼地”。

坚持勤政廉政。结合党的群众路线教育实践活动，大兴落实之风，对全市重大决策部署、重点工作任务，定了就办、说了就干；大兴实干之风，说实话、办实事、求实效；大兴清廉之风，认真执行党风廉政建设责任制，严格落实“一岗双责”，严肃查处各种不正之风和腐败案件；大兴勤俭之风，牢固树立“过紧日子”思想，压缩一切不必要开支，确保“三公”经费只减不增，切实把有限的财力用在刀刃上。

各位代表，新的征程已经开启，新的目标催人奋进。让我们在市委的正确领导下，团结一心，振奋精神，开拓创新，锐意进取，共同创造鸡西人民更加幸福美好的未来!
